# Automated QuantMap for rapid quantitative molecular network topology analysis

**DOI:** 10.1093/bioinformatics/btt390

**Published:** 2013-07-04

**Authors:** Wesley Schaal, Ulf Hammerling, Mats G. Gustafsson, Ola Spjuth

**Affiliations:** ^1^Department of Pharmaceutical Biosciences, Uppsala University, SE-751 24 Uppsala, Sweden and^ 2^Cancer Pharmacology and Computational Medicine, Department of Medical Sciences, Uppsala University, Academic Hospital, SE-751 85 Uppsala, Sweden

## Abstract

**Summary:** The previously disclosed QuantMap method for grouping chemicals by biological activity used online services for much of the data gathering and some of the numerical analysis. The present work attempts to streamline this process by using local copies of the databases and in-house analysis. Using computational methods similar or identical to those used in the previous work, a qualitatively equivalent result was found in just a few seconds on the same dataset (collection of 18 drugs). We use the user-friendly Galaxy framework to enable users to analyze their own datasets. Hopefully, this will make the QuantMap method more practical and accessible and help achieve its goals to provide substantial assistance to drug repositioning, pharmacology evaluation and toxicology risk assessment.

**Availability:** http://galaxy.predpharmtox.org

**Contact:** mats.gustafsson@medsci.uu.se or ola.spjuth@farmbio.uu.se

**Supplementary information:** Supplementary data are available at *Bioinformatics* online.

## 1 INTRODUCTION

Understanding interrelationships between drugs, toxins and other chemicals is an important part of discovering new ways to make better medicines, avoid toxicity or achieve any of a variety of goals for systems chemical biology (Oprea *et al.*, 2007). For many applications, chemicals are commonly compared structurally according to computed or measured properties (Duffy *et al.*, 2012). Another method is to compare the associations with biological systems. One notable application of this method is the connectivity map, which relates biologically active chemicals to gene-expression data (Lamb *et al.*, 2006). Our published QuantMap method explores the connections of chemicals to proteins through protein–protein networks (Edberg *et al.*, 2012).

In this article, we adapt the partially manual QuantMap method to a rapid automated system with an easy-to-use interface, opening up for moderately larger datasets as well as integration with other tools in batch analysis.

## 2 METHODS

### 2.1 Data sources

Chemical to protein relationships were taken from STITCH 3 (Kuhn *et al.*, 2012) through the ‘chemical.aliases.v3.1’ and ‘protein_chemical.links.detailed.v3.1’ tables available from http://stitch.embl.de. Protein to protein relationships (PPI data) were taken from STRING 9.0 (Szklarczyk *et al.*, 2011) through the ‘protein.links.detailed.v9.0’ table available from http://string.embl.de. In both cases, taxonomy was limited to human (designated as 9606) for the current application. MySQL 5.5 (http://www.mysql.com) was used for building the local databases. Chemical identifiers (CIDs) and names generally correspond to PubChem (http://pubchem.ncbi.nlm.nih.gov) but are strictly limited to those found in STITCH 3.

### 2.2 Tools developed

QuantMap automation has been implemented in the Galaxy platform (Blankenberg *et al.*, 2010; Giardine *et al.*, 2005; Goecks *et al.*, 2010) as a separate tool for data preparation and main calculation.

The data preparation tool (named ‘QuantMap Prep’) was written in Perl (http://perl.org) for ease of text processing. This tool checks the list of input chemicals against the local STITCH database. Common names and CIDs known to STITCH are accepted and a table of acceptable CIDs with names is returned. Chemicals that cannot be found will be omitted from the next stage.

The main QuantMap tool (named ‘QuantMap Server’) was implemented in R (http://www.R-project.org/) for access to network analysis tools. QuantMap Server approximates the procedure and parameters described in Edberg *et al.* (2012) and outlined in the Supplementary Figure S1.

For each chemical (CID) cleared in the data preparation step:

1. Retrieve a list of 10 proteins (‘seeds’) closely associated with the current chemical from the local STITCH database. STITCH's overall score with a minimum confidence of 0.7 is used as the measure of association.
2. Retrieve a list of up to 150 proteins associated with these proteins (PPI data) from the local STRING database. STRING's overall score with a minimum confidence of 0.7 is used as the measure of association.
3. Calculate relative importance of the proteins in the PPI network as described in Section 2.3.
4. Condense the importance calculations to a single list ranked by the median of the importance measures.

The ranked lists were combined using Spearman's foot rule (Diaconis and Graham, 1977). This array of pairwise distances was analyzed by hierarchical clustering with hclust (complete) from the STATS package of R. This clustering technique did not require the multidimensional scaling used in the previous work.

### 2.3 Centrality measures (network topology)

In Edberg *et al.* (2012), the relative importance of the proteins (represented as nodes in a network) for a single chemical was modelled by the centrality measures: *betweenness*, *node degree*, *edge percolation component* and *bottleneck betweenness*. These were matched or approximated in the R package igraph (Csardi and Nepusz, 2006) by the functions *degree* (number of connections to a node), *betweenness* (number of shortest paths through a node) and *subgraph.centrality* (number of subgraphs with a node, weighted by size of subgraph) based on computational similarity and rank correlation for a number of test compounds (unpublished data).

## 3 RESULTS

The QuantMap group of tools, encompassing QuantMap Prep and QuantMap Server, are available from the main menu of our Galaxy server (http://galaxy.predpharmtox.org). A list of chemicals identified by name or CID can be loaded into the text field of QuantMap Prep, which checks the names for applicability to the system. QuantMap Server is then run from the result of the preparation job. All chemicals with adequate PPI (according to step 2 of Section 2.2) will be used to produce a plot of the biological interrelationships.

We demonstrate the automated QuantMap system using a dataset identical to the one used by Edberg *et al.* (2012), specifically for Estradiol, Diethylstilbestrol, Tamoxifen, Raloxifene, Fulvestrant, Genistein, Coumestrol, Resveratrol, Bisphenol A, 4-Nonylphenol, Dibutyl phthalate, Zearalenone, Endosulfan, Glimepiride, Rosiglitazone, Aspirin, Ibuprofen and Diclofenac.

**Fig. 1.**
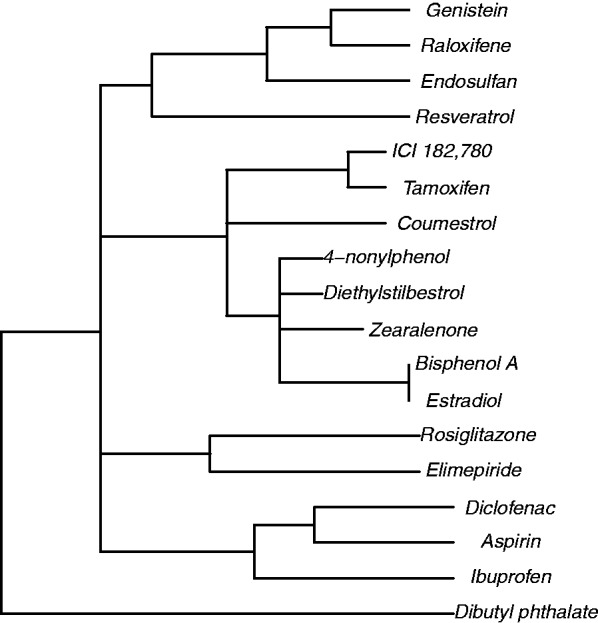
Dendrogram produced by QuantMap Server from the dataset used in this study. The compound list was run through QuantMap Prep to make a data table for QuantMap Server. Compounds separated by fewer branches are more similar to each other from a biological, rather than chemical, perspective. For cleaner display, short branches were flattened to approximate multibranching with di2multi in APE (Paradis *et al.*, 2004)

The dendrogram of Figure 1 shows the results of QuantMap Server applied to this dataset. Qualitatively, this figure is largely equivalent to the results by Edberg, such as the lone dibutyl phthalate and groupings of NSAIDs (Aspirin, Diclofenac and Ibuprofen) and antidiabetic drugs (Glimepiride and Rosiglitazone). The other compounds form a more complex pattern similar, but not identical, to the previous work. Differences could be due to the use of different centrality measures, as described earlier in the text. See Edberg *et al.* (2012) for a general discussion of the results.

The execution time for this analysis was on the order of a few seconds. The speed advantage over a partially manual procedure is considerable. The possibility to create workflows in Galaxy and other types of automation compounds the benefits to help make this automated QuantMap system more practical and accessible.

*Funding*: This work was supported by the Swedish Research Council [VR-2011-6129]; and the Swedish Strategic Research Program eSSENCE.

*Conflict of Interest*: none declared.

## Supplementary Material

Supplementary Data
