# Supplementary material for: Automated QuantMap for rapid quantitative molecular network topology analysis
Source: Bioinformatics. 2013 Jul 4;29(18):2369–70. doi: 10.1093/bioinformatics/btt390 (PMC3753568; doi:10.1093/bioinformatics/btt390)
Supplement: Supplementary Data [file supp_29_18_2369__index.html]

Automated QuantMap for rapid quantitative molecular network topology analysis — Automated QuantMap for rapid quantitative molecular network topology analysis — Automated QuantMap for rapid quantitative molecular network topology analysis — Supplementary Data 

# Automated QuantMap for rapid quantitative molecular network topology analysis

## 

files

**Files in this Data Supplement:**

- Supplementary Data - pdf file
