# Supplementary figures and images for: Automated QuantMap for rapid quantitative molecular network topology analysis
Source: Bioinformatics. 2013 Jul 4;29(18):2369–70. doi: 10.1093/bioinformatics/btt390 (PMC3753568; doi:10.1093/bioinformatics/btt390)

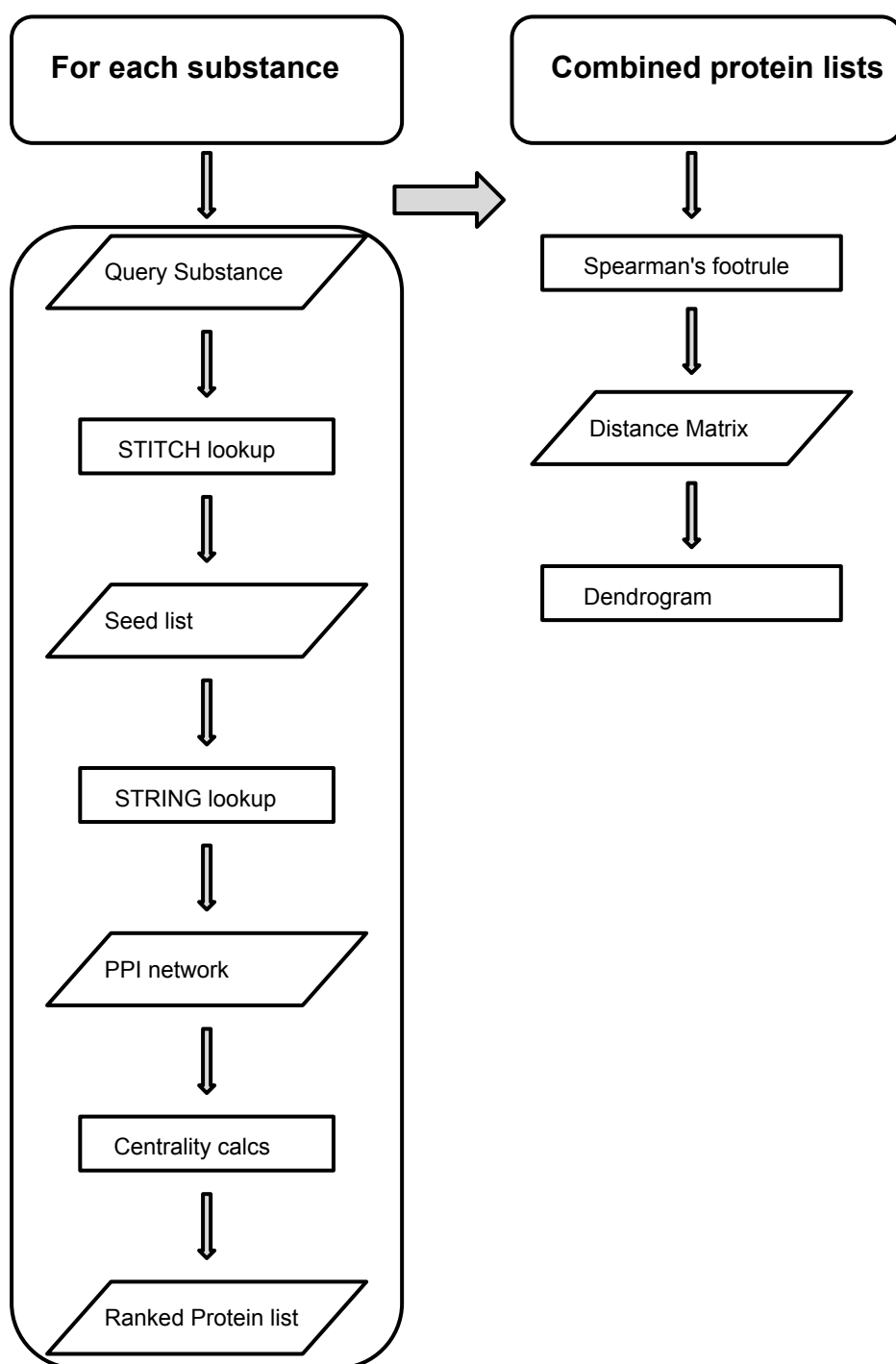

**Supplementary Figure S1.** Flow diagram for QuantMap Calculations.

Supplement: Supplementary Data [file supp_btt390_schaal_quantmap_supplementary-1.pdf]
